# Transient change in GABA_A _receptor subunit mRNA expression in *Lurcher *cerebellar nuclei during Purkinje cell degeneration

**DOI:** 10.1186/1471-2202-7-59

**Published:** 2006-07-27

**Authors:** C Linnemann, I Schmeh, P Thier, C Schwarz

**Affiliations:** 1 Department of Cognitive Neurology, Hertie-Institute for Clinical Brain Research, University of Tübingen, Otfried-Müller-Strasse 27, 72076 Tübingen, Germany

## Abstract

**Background:**

*Lurcher *mice suffer from a complete Purkinje cell (PC) loss in the first four postnatal weeks. Parallel to this degeneration, GABAergic synapses in the deep cerebellar nuclei (DCN), the major recipient of the inhibitory PC projection, increase synaptic conductance. Here, we further investigated this phenomenon, using real-time RT-PCR to assess GABA_A _receptor subunit gene expression during PC degeneration.

**Results:**

We observed a specific reduction in γ2 subunit gene expression, while α1–5, β1–2, γ1,3 and δ subunits were unaffected. We made two further specific findings. First, the difference in gene expression was shown in tissue from DCN only. Neither the hippocampus nor coronal sections through the forebrain showed such effects. Furthermore, the involvement of different levels of corticosterone, a possible humeral trigger for differences in gene expression, could be excluded. Second, like the known potentiation of GABAergic synapses, the γ2 down-regulation was present only after the onset of degeneration at p14. The difference in γ2 mRNA expression, however, appeared transient, since it was no longer detectable in adult *Lurcher *mice.

**Conclusion:**

In conclusion, the down-regulation of γ2 subunits may be related to differences in synaptic efficacy and, as such, may reflect the initial phase of adaptive responses of DCN tissue to massive GABAergic deafferentation. Its transient course, however, does not support the idea that modulations in GABAergic transmission are at the basis of the well-known DCN-based functional benefit of *Lurcher *mice present throughout their life.

## Background

Inhibitory projections are sometimes placed at decisive locations within the motor system and are therefore important for programming and controlling the execution of movement. One of these projections is the GABAergic Purkinje cell (PC) projection onto the deep cerebellar nuclei (DCN) neurons. This projection system undergoes a degeneration in the spontaneous, autosomal semidominant mouse mutation *Lurcher*, yielding nonviable homozygous mice, while heterozygous individuals (*Lc/+*) show ataxia expressed by a tendency to fall [1]. The syndrome is based on a complete loss of Purkinje cells during postnatal development followed by a secondary degeneration of intracerebellar and precerebellar cells [2-4]. The phenotype arises from a mutation in the orphan receptor subunit glutamate δ2 [5] which is expressed almost exclusively in PCs and thus determines the specificity of the primary effect [6]. The first signs of PC degeneration are observed at postnatal day 8 (p8; [7]) and by p26, approximately 90% of PCs have disappeared [2]. The majority of DCN neurons survive, albeit deprived of their inhibitory PC input [8,9].

Behaviorally, *Lc/+ *show deficits in standard tests of motor behavior like the rotarod and other tests of equilibrium [10-15]. However, the symptoms are mild, since differences to wild type animals were seen only in test situations that confront the motor system with higher demands such as, for instance, higher rotation frequencies in the rotarod test [12]. Experiments using lesions of the DCN in *Lc/+ *clearly showed that the mild symptoms reflect a residual functionality of the cerebellar system because lesioned animals showed a grossly deteriorated motor performance in comparison with non-lesioned mutants. Scores measured with the rotarod test dropped virtually to zero without recovery during repeated sessions [16]. Furthermore, spatial orientation, which is mildly impaired in *Lc/+ *[17,18], deteriorated drastically after DCN lesions [19,20]. These behavioral data suggest that the DCN play a beneficial role for the motor performance of *Lc*/+. Electrophysiological recordings in DCN neurons of *Lc/+ *strongly support this hypothesis. *Lc/+ *exhibited a potentiation of GABA_A_-receptor-mediated average synaptic conductance (g_syn_) in close temporal relationship to PC death [21]. This potentiation of g_syn _in the DCN might have an adaptive value, compensating for the loss of the extrinsic inhibition. A possible basis for the modulation of GABAergic synaptic transmission are changes in the subunit composition of GABA_A _receptors [22]. We asked in the present study whether the DCN change their GABA_A _receptor subunit composition in temporal relationship to the degeneration of the inhibitory afferents. Guided by the occurrence of conductance changes between p11 and p14 [21], we used real-time RT-PCR to investigate the gene expression of GABA_A _receptor subunits α1–5, β1–2, γ1–3 and δ in DCN tissue of Lc/+ compared with wild type mice at these critical ages and after the completion of PC degeneration at the age of 8 weeks. Furthermore, we addressed the question as to whether the observed changes are specific to the DCN. In addition, we performed whole cell voltage-clamp recordings of CA1 pyramidal neurons in mutants and controls to determine whether the potentiation in g_syn _previously observed in DCN is a generalized and ubiquitous phenomenon or specific to the denervated DCN.

## Results

Using real-time RT-PCR, mRNA expression levels of GABA_A _receptor subunits α1–5, β1–2, γ1–3 and δ were investigated in tissue of DCN obtained from *Lurcher *mutants aged p11 and p14 and compared to wild type controls of the respective age. Since gene expression of GABA_A _receptor subunits [23] and of internal controls such as hypoxanthine-phosphoribosyltransferase (HPRT) [24,25] are known to be subject to changes during postnatal development, we restricted our comparison of gene expression to age-matched mutants and controls.

**Figure 1:**
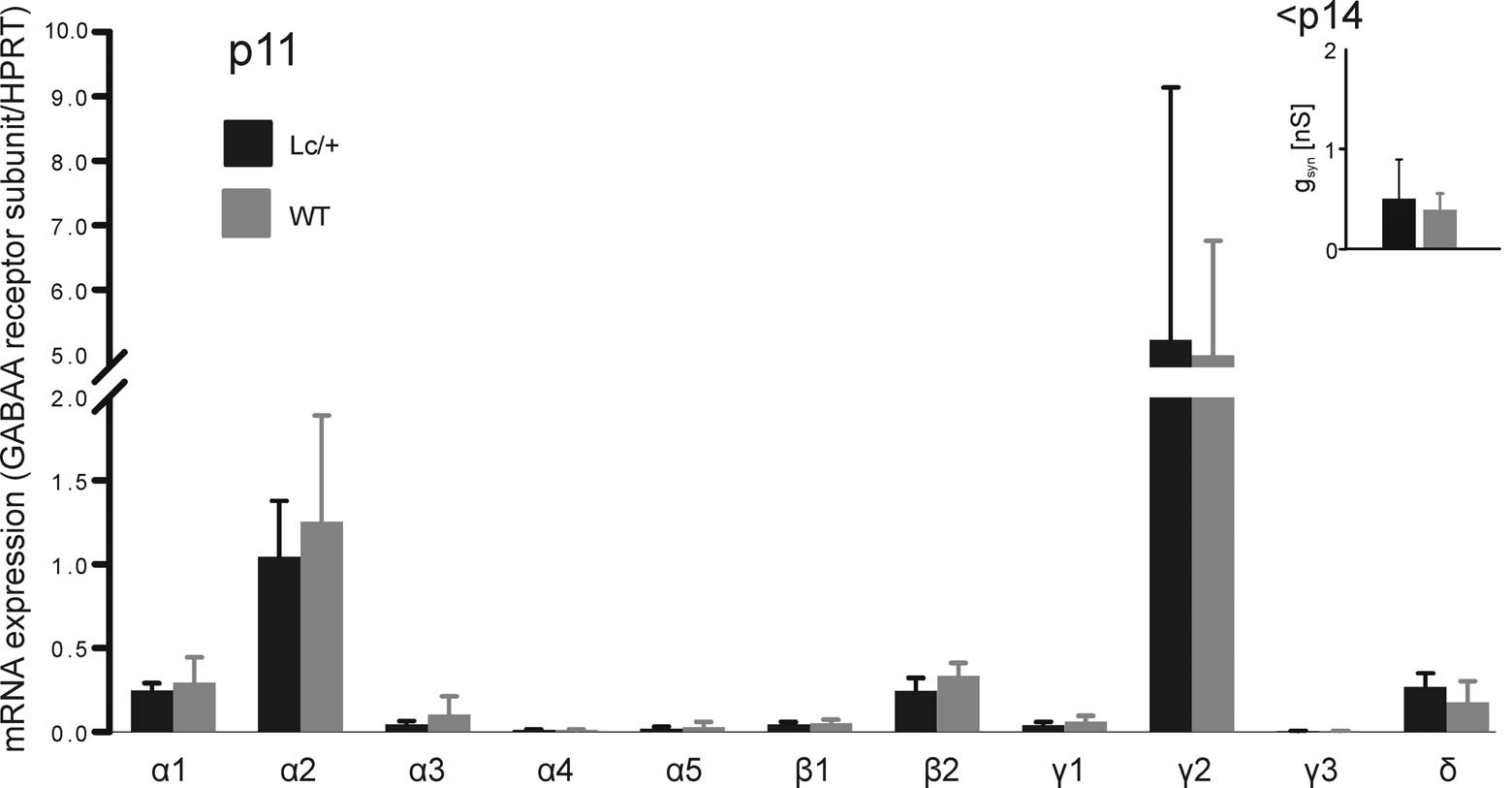
mRNA expression [GABA_A _receptor subunit/hypoxanthine-phosphoribosyltransferase (HPRT)] in DCN tissue of *Lc/+ *(black bars) and wild type mice (WT; grey bars; Mean ± SD) aged postnatal day 11 (p11). No statistically significant differences were found for any of the investigated subunits. The data shown in the inset are from [21] and give the average synaptic conductance (g_syn_) values (with SD as error bars) obtained from mIPSCs recorded from DCN of animals <p14. No statistically significant differences were detected between *Lc/+ *[black bars, 0.50 ± 0.39 nS (n = 3)] and WT [grey bars; 0.39 ± 0.16 nS (n = 4), P = 0.62].

**Figure 2:**
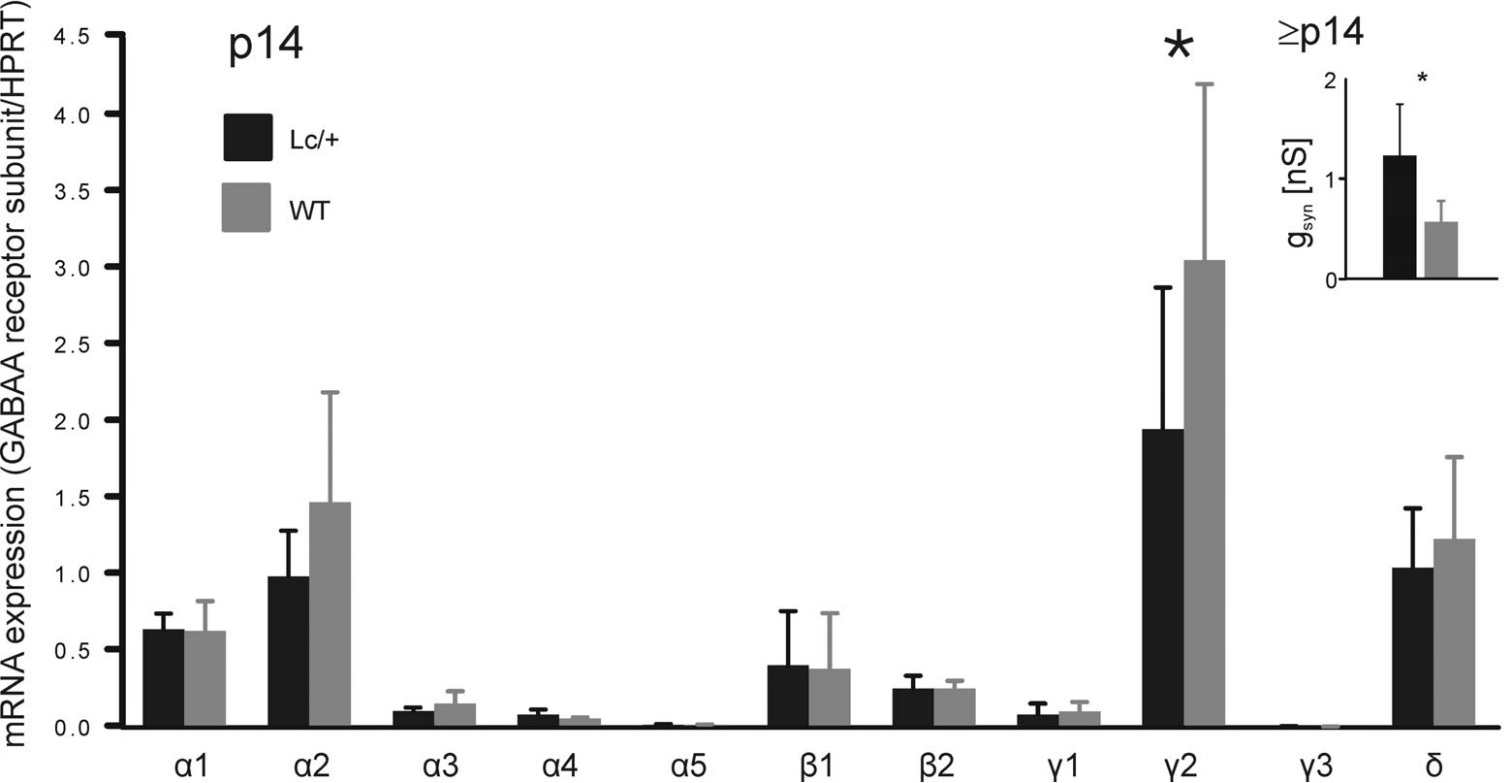
mRNA expression [GABA_A _receptor subunit/hypoxanthine-phosphoribosyltransferase (HPRT)] in DCN tissue of *Lc/+ *(black bars) and wild type mice (WT; grey bars; Mean ± SD) aged postnatal day 14 (p14). *Statistically significant difference for γ2 (n = 9 for mutants/10 for WT; P = 0.036); no statistically significant differences were observed in any of the other investigated subunits. The data shown in the inset are from [21] and give the average synaptic conductance (g_syn_) values (with SD as error bars) obtained from mIPSCs recorded from DCN of animals ≥p14. *Statistically significant difference between *Lc/+ *[black bars, 1.23 ± 0.51 nS (n = 10)] and WT [grey bars; 0.57 ± 0.21 nS (n = 19), *P ≤ 0.001*)].

**Figure 3:**
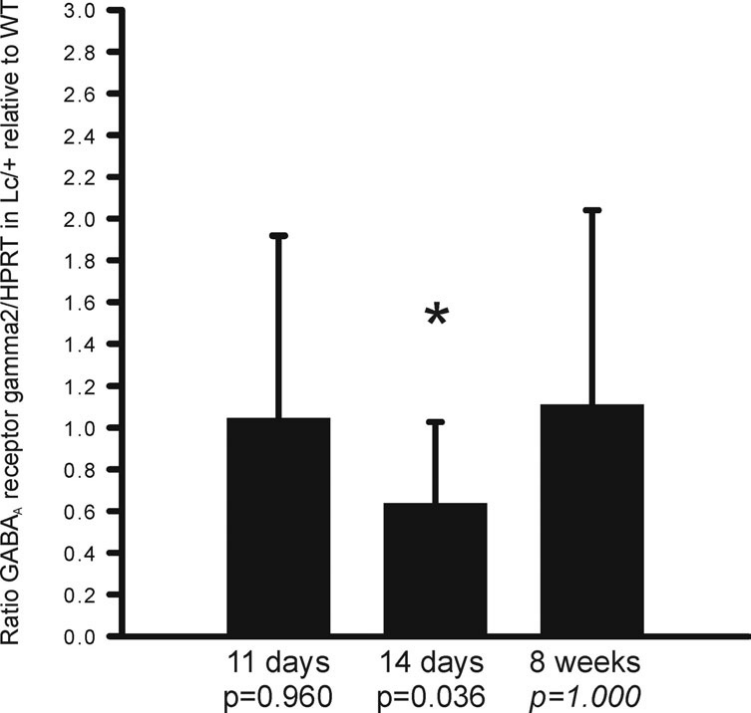
mRNA expression [GABA_A _receptor subunit γ2/hypoxanthine-phosphoribosyltransferase (HPRT)] in DCN tissue of *Lc/+ *relative to wild type mice (WT; Mean ± SD) aged 11 days [n = 5 (*Lc/+*), n = 5(WT)] 14 days [n = 9 (*Lc/+*), n = 10 (WT)], 8 weeks [n = 8 (*Lc/+*), n = 8 (WT)]. *Statistically significant difference between mutants and controls aged 14 days; no statistically significant differences were observed between mutants and controls aged 11 days as well as 8 weeks.

Animals aged p11 showed no clear signs of the characteristic *Lc/+ *phenotype in terms of an ataxic gait or tendency to fall. This time point was also before the onset of changes of g_syn _[21]. In accordance with the lack of differences in behavior and electrophysiological recordings, at p11 we did not find any difference in GABA_A _receptor subunit gene expression between *Lc/+ *and wild type mice either. Figure 1 shows the ratios GABA_A _receptor subunit/HPRT [n = 5 animals each except for wild type α2 (n = 4) and γ3 (n = 3)]. We next investigated the gene expression in the DCN tissue of animals aged p14. At p14, the animals were clearly distinguishable by their mild ataxia, due to the progressive PC degeneration. At this stage, *Lc/+ *exhibit a potentiation of g_syn _in DCN [21]. We observed a statistically significant decrement of the γ2 subunit mRNA expression in *Lc/+ *[1.6-fold down-regulation, ratio GABA_A _receptor subunit/HPRT 1.9390 ± 0.9230 (*Lc/+*, n = 9) versus 3.0410 ± 1.1530 (wild type, n = 10), P = 0.036]. With regard to the other subunits, no differences [n = 5 animals each except for *Lc/+ *α5, β1, γ1 (n = 9) and wild type α5, β1, γ1 (n = 10)] were found. Figure 2 summarizes these results. The decrement of γ2 subunit mRNA expression in *Lc/+ *DCN tissue was of transient nature, since it could not be detected in DCN tissue of eight week old mutants (n = 8) compared to age-matched controls (n = 8, P = 1.000; Figure 3).

**Figure 4:**
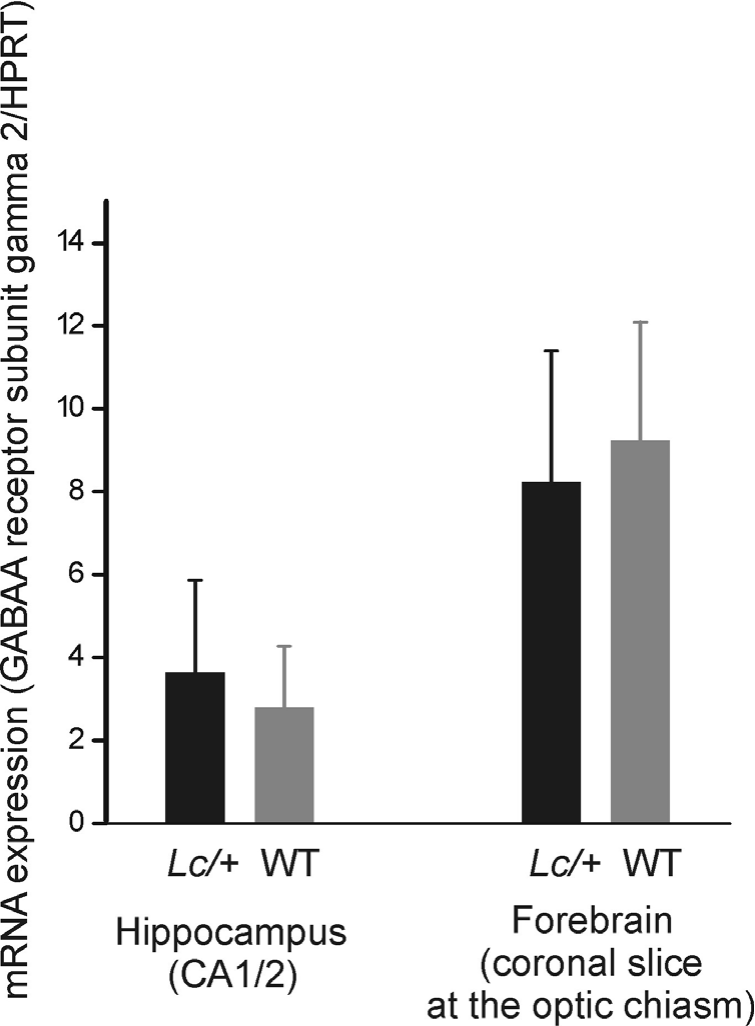
mRNA expression [GABA_A _receptor subunit γ2/hypoxanthine-phosphoribosyltransferase (HPRT)] in hippocampus (CA1/2) and in coronal sections cut through the forebrain at the optic chiasm of *Lc/+ *(black bars) and wild type mice (WT, grey bars, Mean ± SD) aged postnatal day 14. No statistically significant differences were found for hippocampus or for forebrain (coronal slice at the optic chiasm).

**Figure 5:**
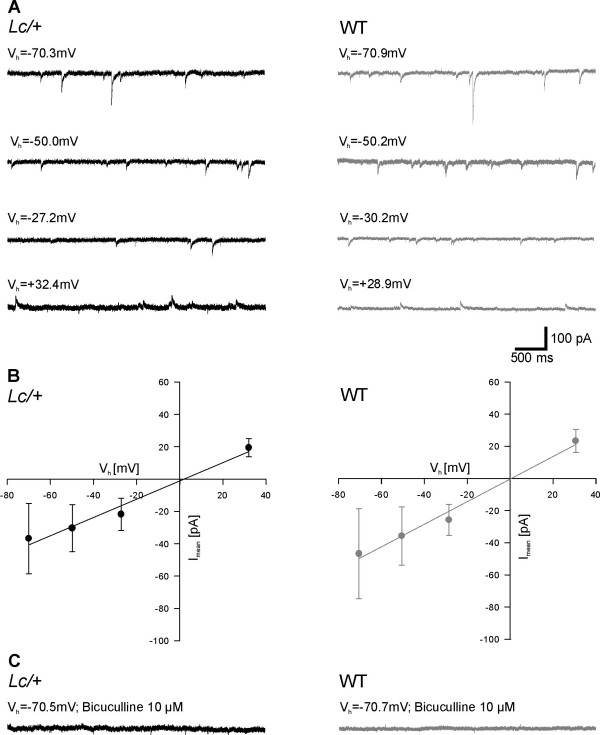
**A:** Recordings of mIPSCs in hippocampal CA1 pyramidal neurons of *Lc*/+ [postnatal day (p) 15] and wild type mice (WT; p17) under chloride symmetrical conditions at the indicated holding potentials (V_h_). **B: **The mean peak amplitudes (I_mean_) of the mIPSCs were plotted versus the V_h _values and a regression line (r^2 ^= 0.97 for *Lc/+*, r^2 ^= 0.98 for WT) was generated to determine the equilibrium potential that was close to the expected 0 mV (+2.2 mV for Lc/+; -1.6 mV for WT). **C: **Application of bicuculline 10 μM blocked the mIPSCs, indicating that they were GABA_A_-receptor-mediated events.

**Figure 6:**
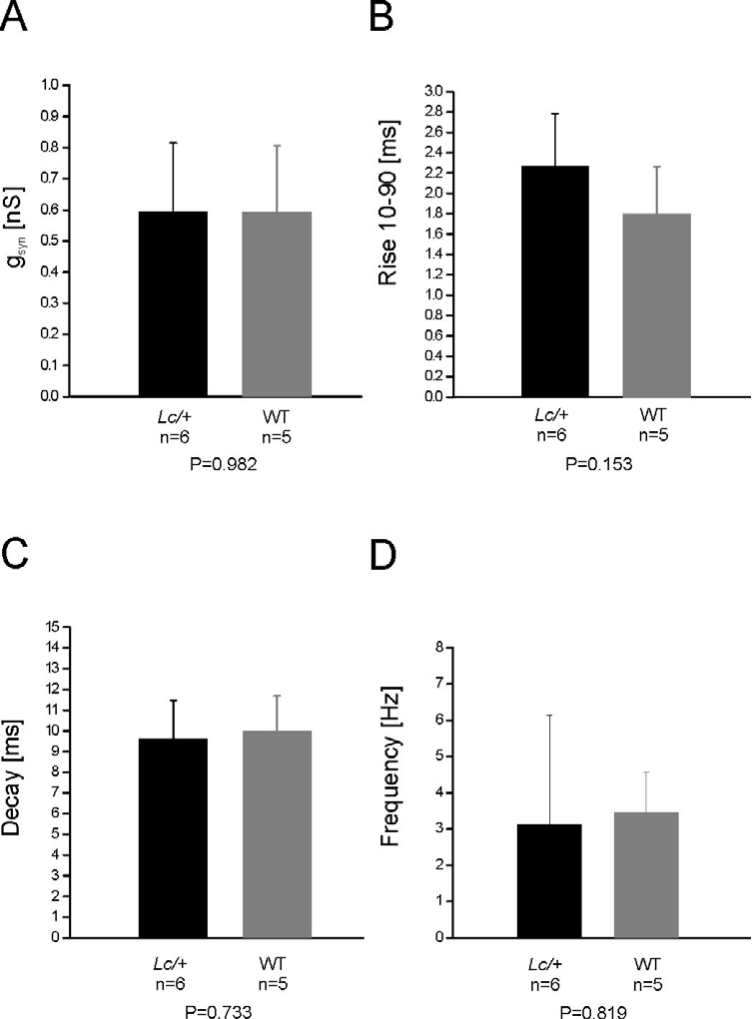
Mean values (with SD as error bars) for the average synaptic conductance (g_syn_, **A**), the rise 10–90 (**B**), the decay times (**C**) and the frequency (**D**) obtained from recordings of mIPSCs in hippocampal CA1 neurons of *Lc/+ *(black bars) and wild type mice (WT, grey bars). Mice were aged p15-18. No statistically significant differences were found for any of the parameters.

Such decrement of γ2 mRNA at p14 was absent in *Lurcher *hippocampus (CA1/2) (n = 5 animals each) and in coronal slices cut through the forebrain at the optic chiasm (n = 5 animals each; Figure 4) – suggesting that the difference in GABA_A _receptor subunit gene expression occurred specifically in the DCN. Furthermore, differences in GABAergic transmission, as found in DCN during PC degeneration [21], were not observed in hippocampal slices taken from mice aged p15–18. Voltage-clamp recordings from hippocampal CA1 pyramidal neurons (n = 6 for mutant neurons, n = 5 for wild type neurons) revealed dense patterns of mIPSCs (Figure 5A, top traces). These were GABA_A_-receptor-mediated events, as judged from the equilibrium potential under chloride symmetrical conditions and pharmacological interference (Figure 5). The equilibrium potentials were close to the expected 0 mV in both mutant and control neurons [+1.8 ± 6.4 mV (*Lc/+*, n = 6); +2.8 ± 3.9 mV (wild type, n = 5), P = 0.758]. The mIPSCs could be blocked regularly in both *Lc/+ *and wild type mice (n = 6 recordings) by application of 10 μM bicuculline, indicating that they were GABAergic (Figure 5C). The average synaptic conductance values were statistically not different between the two groups (Figure 6A). The parameters reflecting the kinetic properties of the mIPSCs (rise 10–90 as well as decay times) did not differ significantly either (Figures 6B an 6C). An analysis of the frequencies of the mIPSCs revealed no differences between mutants and controls (Figure 6D).

**Figure 7:**
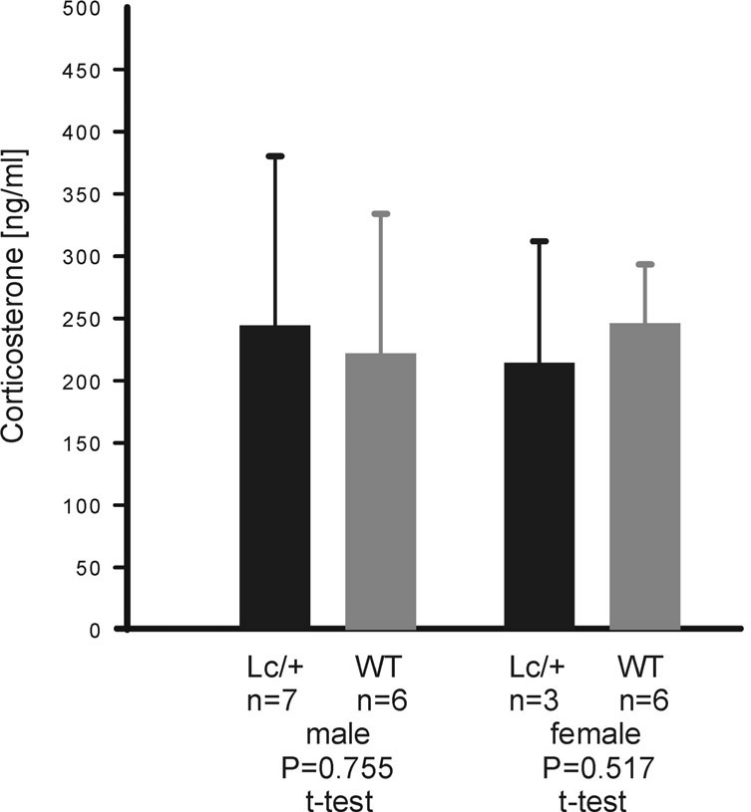
No statistically significant differences were found in corticosterone serum levels in *Lc/+ *and wild type mice (WT) aged postnatal day 14. Corticosterone serum levels were measured as described in the Methods section. n indicates the number of animals.

To rule out the possibility that different corticosterone levels at the time of animal preparation might affect our electrophysiological and/or gene expression results, serum corticosterone levels were measured in mutant and control mice aged p14. To avoid variations due to sex differences, we analyzed the data obtained from male and female mice separately. We found no differences in corticosterone serum level between mutants and controls (Figure 7).

## Discussion and conclusion

The residual motor functionality of *Lurcher *mice is based on the intactness of the DCN and outlasts the period in which PCs degenerate [16]. To elucidate the role of candidate cellular mechanisms for this compensation, their specificity for the structure (DCN) and time of occurrence (degeneration of PCs) must be investigated. Mechanisms showing no clear specificity and relationship to the DCN (or connected structures) or to the occurrence of the PC degeneration can be excluded as a basis for this functional compensation. In a previous study from our laboratory, *Lurcher *mice displayed a potentiation of miniature IPSCs in the DCN in close temporal relationship to PC degeneration [21]. Our present data complement these earlier results by showing that the synaptic conductance of GABAergic IPSCs is unaffected in another brain structure, the hippocampus.

The responsiveness of the hypothalamo-pituitary-adrenal system is known to be enhanced in *Lurcher *mice. Under stress, this mutant shows increased levels of serum corticosterone compared to wild type controls [26,27]. Corticosterone has a known modulatory role for GABA_A_-receptor-mediated conductance [28] as well as GABA_A _receptor subunit gene expression [29,30]. The absence of significant differences of serum corticosterone between *Lurcher *and wild type mice (at the ages and under the experimental conditions investigated here), thus supports the idea that the changes observed in DCN of *Lurcher *mice are not unspecific phenomena that occur throughout the brain. The results instead suggest that these changes are specific to the DCN. The down-regulation of γ2 mRNA matches the spatiotemporal specificity of the electrophysiological changes in GABAergic transmission. This was detected in the DCN, but not in material taken from the hippocampus or from a section through the forebrain. Furthermore, its de novo occurrence at p14 indicates a temporal relationship to the course of PC degeneration. However, since it was not detectable in adulthood (eight weeks of age), the reduction of subunit gene expression cannot be the direct structural cause of the permanent mild expression of ataxia in *Lurcher *mice. If future work is to show a functional link between subunit composition and synaptic potentiation, the latter must be subjected to the same reasoning and must be excluded from the candidates of mechanisms directly responsible for functional adaptation in the DCN. The main question to be discussed below is therefore, to what extent can a changed subunit expression relate to changes in synaptic transmission in the *Lurcher *mouse.

First and foremost, the differences in gene expression were calculated with reference to the ubiquitously expressed HPRT. Therefore, the measurements should be insensitive to known differences of volume and cell densities in *Lurcher *DCN [8,9]. This may help to explain apparent conflicting results obtained in adult *Lurcher *mice using in situ hybridization, where increased γ2 mRNA levels in *Lurcher *mice aged p108 based on the assessment of autoradiographic grain density were reported [31]. The different results may be partly explained by the different ages of animals and the different methods used, but the shrinkage of the DCN (confere [8,9]) may well have contributed to an increase in grain density (as discussed in [31]). The insensitivity of the real-time RT-PCR to shrinkage of the DCN is further supported by the exclusive reduction of the γ2 subunit mRNA expression. If general differences in cell densities, volume and presence of GABAergic synapses in DCNs of *Lurcher *and wild type mice had accounted for differences in mRNA expression, the consequences should have affected all, or at least many, subunits. Our findings imply that the relative composition of postsynaptic GABA_A _receptors during the time of PC degeneration was different in *Lurcher *mice. This conclusion is in line with the probable postsynaptic location of the mechanism, leading to the increment in miniature synaptic conductance [21]. There are two possible explanations as to how the present findings may be related to changes in synaptic conductance. First, it is well established that the compositions of subunits determine single channel conductances [32-35]; see for review [22]. Second, subunit composition specifically affects the dynamic control of GABA_A _receptor expression at the cell surface and may thus effectively determine its presence at the postsynaptic membrane ([36-39]; see for review also [40].

If modulation of single channel conductances were at the basis of the synaptic potentiation, then our present data, together with earlier results, [21] would imply that a reduced γ2 content increases channel conductance and hence synaptic conductance. This prediction, however, would be in conflict with previously published data of several investigators. Verdoorn et al. [32] analyzed GABA-gated chloride channels expressed in human embryonic kidney cells. When the γ2 subunit was present, the resulting GABA_A _receptors had a larger conductance. Moreover, Lorez et al. [35] investigated the channel properties of neuronal GABA_A _receptors from brains of embryonic and postnatal mice lacking the γ2 subunit and found smaller single channel conductances. To rescue this scenario, one would need to assume that the decrement of the γ2 subunit mRNA expression found here is accompanied by the replacement of γ2 by another subunit that has the potential to increase the g_syn_, but that has not been detected so far. Replacements for GABA_A _receptor subunit composition during postnatal development under physiological conditions are well known. For instance, α2 subunits are partially replaced by α1 subunits in DCN during the first two postnatal weeks [23]. Such switches have indeed the potential to change the synaptic strength of GABA_A _receptors [41,42].

The second possibility is that subunit composition is altered to dynamically regulate the receptor availability at the cell surface. DCN neurons in rodents express mainly α1, β2 and γ2 subunits [31,43]. The presence of the α1 subunit appears to be required for the surface expression of β2 (as investigated in Madin-Darby canine kidney cells [37] and in COS7 cells [36]). Our study shows that the content of α1 and β2 mRNA remained unchanged in *Lurcher *mice compared to the age-matched controls. None of these facts suggest that the amount of generated GABA_A _receptors and their subsequent transport to the cell membrane is impaired in *Lurcher *mice. On the other hand, it may also be possible to regulate receptor presence by interfering with the removal of receptors from the membrane. GABA_A _receptors undergo a constitutive endocytosis after association with the adaptin AP2 complex, a process dependent on the presence of β and γ subunits [39]. Moreover, blockade of endocytosis enhances the amplitude of GABA_A_-mediated mIPSCs in hippocampal neurons [39]. More specifically, Connolly et al. [38] ascertained that cell surface stability of GABA_A _receptors depends on the presence of γ2 subunits. They observed that receptors containing γ2 are selectively removed from the cell surface upon activation of protein kinase C with concomitant reduction of GABA-induced currents. In summary, a tentative hypothesis – to be tested by further experiments – can be formulated from these considerations as follows: The specific γ2 reduction of subunit expression during PC degeneration in *Lurcher *mice might modulate endocytosis such as to allow the *Lurcher *DCN neurons to specifically increase the GABA_A _receptor cell surface levels at the postsynaptic membrane. This speculation receives some experimental support from the results of a non-stationary fluctuation analysis on mIPSCs in wild type and *Lurcher *mice. This analysis gave no indication of changes of single channel conductances, while the peak number of open receptors underlying *Lurcher *mIPSCs was estimated to be of a factor two higher than in wild type mice [21].

## Methods

### Animals and molecular genotyping

Heterozygous B6CBACa-A^w-J^/A-*Lc *(Jackson Laboratories, Bar Harbor, ME, USA) were mated with B6CBA (Harlan-Winkelmann, Borchen, Germany) mice. The animals were kept and used in experiments according to the institutional and national animal care guidelines (also conforming with NIH guidelines on animal care). Animals from the *Lurcher *progeny not showing the characteristic *Lc*/+ phenotype (ataxic gait and tendency to fall) were genotyped as described previously [21].

### Real-time RT-PCR

Following deep anesthesia by application of ketamine (150 mg/kg i.p.), the brain was removed and prepared as follows under RNase-free conditions. DCN tissue was dissected from parasagittal cerebellar slices (400–500 μm thickness). Hippocampal CA1/2 tissue was isolated from horizontal cerebral slices (400–500 μm thickness). Forebrain slices were obtained from 1000–1200 μm thick coronal slices, cut through the forebrain at the optic chiasm. The tissue samples were immediately transferred into RNA *later *RNA Stabilization Reagent (Qiagen, Hilden, Germany) to stabilize and protect RNA in tissue. Total RNA was isolated using RNeasy Mini Kit (Qiagen) including a rotor-stator homogenization (Ultra Turrax T8, IKA-Werke, Staufen, Germany) of the samples. All samples were treated with DNase (DNA-*free*, Ambion, Huntingdon, United Kingdom) and tested for contaminating genomic DNA by PCR using a non-intron spanning primer pair for β-actin (forward 5'-cacccgccaccagttcgcca-3', reverse 5'-caggtcccggccagccaggt-3'; MWG-Biotech, Ebersberg, Germany). First-strand cDNA was prepared from total RNA by reverse transcription using the Superscript II RNase H^- ^Reverse Transciptase (Invitrogen, Karlsruhe, Germany) and random hexamers. Gene expression was measured in the ABI Prism 7700 Sequence Detection System (Applied Biosystems, Foster City, California, USA). Primers (MWG-Biotech, Ebersberg, Germany) were selected to result in amplicons less than 150 bp to increase the efficiency of PCR amplification. The sequences of the primers are listed in Table 1. Product specificity of the PCR products was confirmed by dissociation curve analysis and/or agarose gel electrophoresis. Amplification of specimens and serial dilutions of standards (PAGE-purified oligonucleotides; Thermo Electron, Ulm, Germany) was carried out in a total volume of 15 μl including Sybrgreen Master Mix (Applied Biosystems) and primers (final concentration 300 nM) under the following conditions: 50°C for 2 min; 95°C for 10 min; 40 cycles with 95°C for 15 s and 60°C for 1 min. Standard curve extrapolations were calculated for the genes of interest to obtain the quantity of starting amount for each sample. All results were obtained from duplicates and normalized with respect to the internal control HPRT to correct for sample to sample variations.

### Electrophysiology

Mice aged p15-18 (without statistically significant age differences between mutants and controls) were deeply anesthetized by application of ketamine (150 mg/kg i.p.). The brains were removed and placed in ice-cold modified artificial cerebrospinal fluid [ACSF, containing (in mM) 126 sucrose, 2.5 KCl, 1.3 NaH_2_PO_4_, 3 MgCl_2,_, 26 NaHCO_3_, 0.1 CaCl_2 _and 20 D-glucose] that was bubbled with 95%O_2_-5%CO_2_. Horizontal slices of 275 μm thickness that included the hippocampus were obtained using a vibratome (VT1000s, Leica, Nussloch, Germany) and transferred into modified ACSF at room temperature. The modified ACSF was replaced within the next 90 min with ACSF containing (in mM) 125 NaCl, 2.5 KCl, 1.3 NaH_2_PO_4_, 2 MgCl_2,_, 26 NaHCO_3_, 2 CaCl_2 _and 20 D-glucose, oxygenated with 95%O_2_-5%CO_2 _at room temperature. Experiments were performed with this ACSF to which the selective non-NMDA receptor antagonist 6,7-dinitroquinoxaline-2,3-dione (25 μM; Tocris, Bristol, UK), tetrodotoxine (1 μM, Tocris) and, in some recordings, also the GABA_A _antagonist bicuculline (10 μM; Sigma, Taufkirchen, Germany) were added.

Whole-cell voltage-clamp recordings of hippocampal CA1 pyramidal neurons were conducted with patch pipettes that had a resistance of 2.5–3.5 MΩ when filled with a solution containing (in mM) 124 CsCl, 10 K^+^-HEPES, 5 EGTA, 4.6 MgCl_2_, 4 K^+^ATP, 0.4 Na^+^GTP, 0.1 CaCl_2_, and 5 QX-314 (Tocris), adjusted to pH 7.3 with CsOH, thus obtaining chloride symmetrical experimental conditions. CA1 pyramidal neurons were identified and the patch procedure was visualized using a motorized (Luigs&Neumann, Ratingen, Germany) microscope (Axioscope, Zeiss, Göttingen, Germany) with water immersion objective (×40, Zeiss numerical aperture 0.75), infrared illumination, Normaski optics and infrared sensitive CCD camera (Newvicon C2400-07-C Hamamatsu, Japan). The recordings were performed with an EPC-7 amplifier (Heka, Lambrecht, Germany). The data was sampled at a rate of 25 kHz (Spike2, CED, Cambridge, UK). Neurons were voltage-clamped at -70.6 ± 0.7 mV. Data was not quantitatively analyzed if, under these conditions, the serial resistance (17.9 ± 7.3 MΩ, n = 11) had changed by more than 20% or if the membrane resistance (221.8 ± 66.8 MΩ, n = 11) of the recorded neuron was below 150 MΩ. Neither the membrane nor the serial resistances differed significantly between genotypes.

Analysis of the miniature inhibitory postsynaptic currents (mIPSCs) was performed with the MiniAnalysis program (Synaptosoft, Decatur, GA, USA). For analysis of the synaptic waveforms, only those synaptic events that had not been interrupted by other synaptic events were evaluated. g_syn _values were calculated as g_syn _= I_mean_/(V_h _- E), where I_mean _is the mean peak amplitude of the mIPSCs, V_h _is the holding potential and E is the measured equilibrium potential for the mPSCs of the respective recording. The decay time mentioned in this paper is the time it takes the current values to decay to 1/*e *of the peak values of the mIPSC amplitudes.

### ^125^I radioimmunoassay

Following deep anesthesia by application of ketamine (150 mg/kg i.p.), blood was collected by intracardial puncture and immediately transferred into a plasma separator tube filled with lithium heparin (BD Microtainer PST LH, Becton Dickinson, Heidelberg, Germany) on ice. After centrifugation, the sera were stored at -20°C until the ^125^I radioimmunoassay was performed. The Coat-A-Count Rat Corticosterone (TRKC 1; DPC Biermann, Bad Nauheim, Germany) procedure is a solid-phase ^125^I radioimmunoassay. ^125^I-labelled rat corticosterone competed for 2 h at room temperature with corticosterone in the serum specimens for antibody sites immobilized to the wall of the tubes. The counts per minute measured in the tubes with a gamma counter (Multi-Crystal Counter LB 2104, Berthold, Bad Wildbad, Germany), converted by way of a calibration curve yielded to a measure of the corticosterone serum level. The minimal detectable dose is approximately 5.7 ng/ml. The blood collections were always performed in the same time window in the afternoon, to avoid variations due to circadian rhythm.

### Statistical analysis

Statistically significant differences and correlations were tested with the Student's t-test for normally distributed data, and otherwise with the Mann-Whitney-Test (italicized P values throughout the paper). Statistical significance was assumed if P < 0.05.

## Authors' contributions

CL, PT and CS designed and analyzed the experiments and drafted the manuscript. CL and IS carried out the experiments. All authors read and approved the final manuscript.

## Figures and Tables

**Table 1:** Primers for GABA_A _receptor subunits and the internal control

	forward	reverse
GABA_A _alpha1	5'-gccccgagctgtgcaa-3'	5'-ggtccaggcccaaagatagtc-3'
GABA_A _alpha2	5'-acagtccaagccgaatgtcc-3'	5'-aacggagtcagaagcattgtaagt-3
GABA_A _alpha3	5'-aacaaatctgtggaagtagcacagg-3'	5'-tctctgtcccaacaacatggcc-3'
GABA_A _alpha4	5'-ataagtgcggagtgccccatg-3'	5'-tcccaaatttcaaagggcag-3'
GABA_A _alpha5	5'-gcagcttgaggactttccaatg-3'	5'-ggtaagcataactgccaaatttca-3'
GABA_A _beta1	5'-accagagtgccaatgaaaagaac-3'	5'-gctgaggagaatattgccgtg-3'
GABA_A _beta2	5'-ccacatccgaagcagtaatggg-3'	5'-tgctggaggcatcataggc-3'
GABA_A _gamma1	5'-cactccttgtactcactgtgtggt-3'	5'-tccttcctccactagctcagtcc-3'
GABA_A _gamma2	5'-caaatacatggagcattggaagc-3'	5'-tcatagtcatcatctgacttttggc-3'
GABA_A _gamma3	5'-ttcagttgaggcagctgatca-3'	5'-ctgaggcccatgaagtcaaac-3'
GABA_A _delta	5'-gtggccagcattgaccatatc-3'	5'-tctgatgcaggaacacagtcatg-3'
HPRT	5'-gcctaagatgagcgcaagttg-3'	5'-tactaggcagatggccacagg-3'
